# Failure of High-Flow Nasal Cannula Therapy in Pneumonia and Non-Pneumonia Sepsis Patients: A Prospective Cohort Study

**DOI:** 10.3390/jcm10163587

**Published:** 2021-08-15

**Authors:** Eunhye Kim, Kyeongman Jeon, Dong Kyu Oh, Young-Jae Cho, Sang-Bum Hong, Yeon Joo Lee, Sang-Min Lee, Gee Young Suh, Mi-Hyeon Park, Chae-Man Lim, Sunghoon Park

**Affiliations:** 1 Department of Pulmonary, Allergy and Critical Care Medicine, Hallym University Sacred Heart Hospital, Anyang 14068, Korea; eunhye94@hallym.or.kr; 2 Samsung Medical Center, Department of Critical Care Medicine, Sungkyunkwan University School of Medicine, Seoul 06351, Korea; kjeon@skku.edu (K.J.); suhgy@skku.edu (G.Y.S.); 3 Asan Medical Center, Department of Pulmonary and Critical Care Medicine, University of Ulsan College of Medicine, Seoul 05505, Korea; synthesis83@hanmail.net (D.K.O.); sbhong@amc.seoul.kr (S.-B.H.); yeagi7@gmail.com (M.-H.P.); cmlim@amc.seoul.kr (C.-M.L.); 4 Department of Pulmonary and Critical Care Medicine, Seoul National University Bundang Hospital, Seongnam 13620, Korea; lungdrcho@gmail.com (Y.-J.C.); yjlee1117@snubh.org (Y.J.L.); 5 Department of Pulmonary and Critical Care Medicine, Seoul National University Hospital, Seoul 03080, Korea; sangmin2@snu.ac.kr

**Keywords:** high flow nasal cannula, intubation, outcomes, sepsis

## Abstract

Despite the increasing use of high-flow nasal cannulas (HFNCs) to treat critically ill patients, data on their effectiveness for sepsis patients remains very limited. We studied a prospective cohort of sepsis patients from the Korean Sepsis Registry (18 intensive care units (ICUs)). Patients started on HFNC therapy for hypoxemia within the first three ICU days were enrolled. HFNC failure was defined as intubation or ICU death, and the primary outcome was early HFNC failure occurring within 72 h of HFNC initiation. Of 901 patients with sepsis admitted to the ICU, 206 who received HFNC therapy were finally included (117 with pneumonia vs. 89 with non-pneumonia sepsis; median age, 71.0 (63.0–78.0) years; P_a_O_2_/F_i_O_2_ ratio, 160.2 (107.9–228.2) mm Hg; septic shock, *n* = 81 (39.3%)). During HFNC therapy, 72 (35.0%) patients were intubated and 51 (24.8%) died. HFNC failure developed in 95 (46.1%) patients, and among them, early failure rate was 85.3% (81/95). On multivariate analysis, an immunocompromised state (odds ratio (OR) = 2.730), use of a combination of antibiotics (OR = 0.219), and the P_a_O_2_/F_i_O_2_ ratio (OR = 0.308) were significantly associated with early HFNC failure in pneumonia sepsis patients. However, in non-pneumonia sepsis patients, lactate levels (OR = 1.532) were significantly associated with early HFNC failure. In conclusion, a high proportion of sepsis patients experience HFNC failure, usually within 72 h after therapy initiation, which emphasizes the importance of close monitoring. Furthermore, unlike in pneumonia sepsis, organ failure (i.e., lactate) might serve as a prognostic marker in non-pneumonia sepsis (i.e., type IV respiratory failure).

## 1. Introduction

Sepsis is a life-threatening infectious condition and imposes a substantial global health burden [1,2,3]. With regard to the initial treatment, in addition to hemodynamic resuscitation and early antibiotics, oxygen therapy is important for sepsis patients exhibiting hypoxemia. Recently, as a noninvasive strategy for acute hypoxemic respiratory failure (AHRF), use of high-flow nasal cannulas (HFNCs) has become popular, as their use can decrease the need for intubation [4,5]. It may also reduce the work required to breathe and improve cardiovascular dynamics in critically ill patients [6,7,8].

A recent multicenter randomized study showed that HFNC was associated with lower mortality and a lower risk of intubation compared to noninvasive ventilation or standard oxygen therapy in AHRF patients, including those who were immunocompromised [4,5]. The respiratory rate–oxygenation (ROX) index was also developed based on a large cohort study to predict the success of HFNC therapy in patients with pneumonia [9]. However, most previous studies examined its role in primary respiratory failure (i.e., type I respiratory failure) and not in sepsis or septic shock, where there are issues of oxygen delivery and oxygen utilization at tissue level, as well as lung injury, secondary to the uncontrolled inflammatory process (i.e., type IV respiratory failure). Hence, we hypothesized that HFNC outcomes and their risk factors would be different in sepsis patients, compared to those with primary respiratory failure.

In the present study, we investigated the rates of HFNC failure and analyzed the risk factors associated with HFNC failure in a prospective cohort of pneumonia and non-pneumonia sepsis patients.

## 2. Methods

### 2.1. Study Population

This prospective cohort study analyzed data from the Korean sepsis registry. Eighteen ICUs of 17 tertiary or university-affiliated hospitals that run educational programs on sepsis bundles participated in the study. We analyzed data obtained over the 6-month period from September 2019 to February 2020. To verify data quality, regular audits were conducted by research committee members. All consecutive patients admitted to ICUs with diagnoses of sepsis or septic shock were screened for eligibility. All patients were followed-up until the date of death or hospital discharge. The inclusion criterion was HFNC therapy to treat hypoxemia during the first 3 ICU days. For all patients, the fraction of inspired oxygen (F_i_O_2_) and flow rate (L/min) were adjusted at the discretion of the physician to maintain an S_p_O_2_ > 92%. The exclusion criteria were non-admission to an ICU, initiation of HFNC therapy 72 h after ICU admission, mechanical ventilation (MV) before HFNC therapy (e.g., post-extubation HFNC), incomplete P_a_O_2_/F_i_O_2_ ratio data on the day of HFNC initiation, and no data on hospital outcomes. The study was approved by the institutional review boards of all participating hospitals, including the Hallym University Institutional Review Board (approval no. 2018-09-004). Given the observational nature of the study, the requirement for written informed consent from patients or their legal surrogates was at the discretion of the ethics committees of the participating hospitals. We followed the STROBE guidelines for reporting of observational cohort studies [10].

### 2.2. Data Collection

The study coordinators at each participating hospital prospectively collected data using an electronic case report form (http://sepsis.crf.kr/; accessed on 1 January 2021). The following information was recorded: demographic data (including age, sex, and comorbidities); physiological and laboratory parameters and the Simplified Acute Physiology Score 3 (SAPS3) at ICU admission [11]; the P_a_O_2_/F_i_O_2_ ratio, lactate levels, and SOFA scores [12] on the day of HFNC initiation (i.e., the pre-HFNC values); infection source and type (i.e., community- or hospital-acquired); multidrug-resistant (MDR) pathogen status in patients with positive culture results; the adequacy of empirical antibiotic therapy; the rate of compliance with the 3-h Surviving Sepsis Campaign bundle [13]; treatments during the first 3 ICU days (transfusion, steroid therapy, noninvasive ventilation, and continuous renal replacement therapy (CRRT)); the net fluid balance during the pre-ICU period and on ICU day 1; and outcome data (including intubation (i.e., MV treatment) and ICU and in-hospital mortality). All information was anonymized.

Community-acquired infection was defined as an infection that occurred in a community setting; hospital-acquired infection was defined as an infection that developed no earlier than 48 h after hospitalization. The culprit pathogen was defined as any agent cultured from samples collected within 48 h or at the time of sepsis diagnosis. The adequacy of empirical therapy was determined based on drug susceptibility testing or the recommendations of relevant guidelines [14,15]. MDR was defined as a microorganism resistant to agents from at least three antimicrobial categories [16].

### 2.3. Diagnosis of Sepsis and Septic Shock

The Sepsis-3 criteria were used to diagnose sepsis and septic shock [17]. First, we screened patients with suspected infections using the quick SOFA score. If the score was ≥2, organ dysfunction was assessed using the full SOFA score. The criteria for diagnosing sepsis included a probable or confirmed infection, and a change in the total SOFA score of ≥2 after infection. Septic shock was defined as persistent arterial hypotension requiring a vasopressor to maintain a mean arterial pressure of ≥65 mmHg, and a serum lactate level >2 mmol/L, despite adequate volume resuscitation.

Time zero was defined as the time of triage in the emergency department (ED) for patients who presented to an ED, or the time of sepsis diagnosis by a physician or nurse for those in general wards. The 3-h sepsis bundle completion rates (i.e., compliance) were measured based on time zero (Supplemental Materials) [13,18]. Any bolus infusion of crystalloid fluid was considered to indicate compliance with the fluid bundle component.

### 2.4. Data Analyses

HFNC failure was defined as intubation or death in the ICU (whichever occurred first; the composite outcome). The primary endpoint was the rate of early HFNC failure (within 72 h of initiation of HFNC therapy). We also aimed to identify factors significantly associated with early HFNC failure; this analysis was performed separately for pneumonia and non-pneumonia sepsis patients.

Categorical variables are presented as numbers (%), and continuous variables as medians (interquartile ranges, IQRs). To compare continuous variables, the Mann–Whitney *U* test was used, and for categorical variables, the chi-square or Fisher’s exact test was used. For multivariable analyses, logistic regression analyses were performed using covariates with a *p* value of <0.05 on univariable analyses; a backward stepwise selection method based on the likelihood ratio was employed, and variables with overlapping meaning were removed in the model.

A meta-analysis showed that 24.3% of patients with acute hypoxemic respiratory failure experienced HFNC failure (i.e., intubation) [19], and based on our previous retrospective sepsis study [20], the rate of ICU death was expected to be 33.0%. Hence, with a power of 80% and a type I error rate of 5% (two-sided), the calculated sample size was 202 patients in the present study. IBM SPSS for Windows software (ver. 25.0; IBM Corp., Armonk, NY, USA) was used for all statistical analyses. A *p* value <0.05 was considered significant.

## 3. Results

### 3.1. Study Population

**Figure 1:**
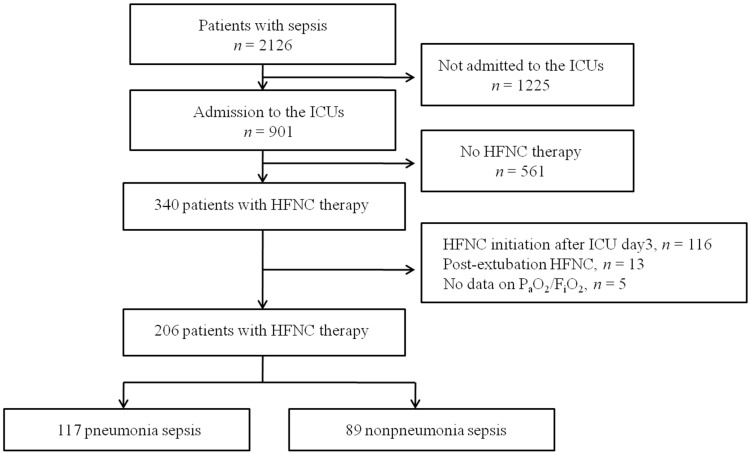
Flow chart of patient enrolment. HFNC: high-flow nasal cannula; ICU: intensive care unit.

Of 2126 patients who met the Sepsis-3 criteria during the 6-month study period, 901 were admitted to ICUs and screened for study eligibility. After excluding patients who met the exclusion criteria, 206 patients (117 with pneumonia sepsis and 89 with non-pneumonia sepsis) were finally enrolled (Figure 1). The median age was 71.0 years (interquartile range: 63.0–78.0 years), and 64 (31.1%) were women (Table 1). The median P_a_O_2_/F_i_O_2_ at HFNC initiation was 160.2 (107.9–228.2) mm Hg; 81 patients (39.3%) were in septic shock. Diabetes (35.4%) and an immunocompromised state (i.e., a hematological malignancy, solid cancer, or drug-induced immunosuppression; 34.5%) were the most common underlying comorbidities. The prevalence rates of bacteremia and MDR pathogens were 24.8% and 19.4%, respectively; hospital-acquired infections were present in 36.9% of patients. As shown in Table 1, initial disease severity was higher in patients with non-pneumonia sepsis than in those with pneumonia sepsis. The lactate levels and illness severity (SOFA scores and SAPS3), and the rates of septic shock and bacteremia, were higher in the non-pneumonia sepsis patients.

### 3.2. Treatments and Outcomes

**Figure 2:**
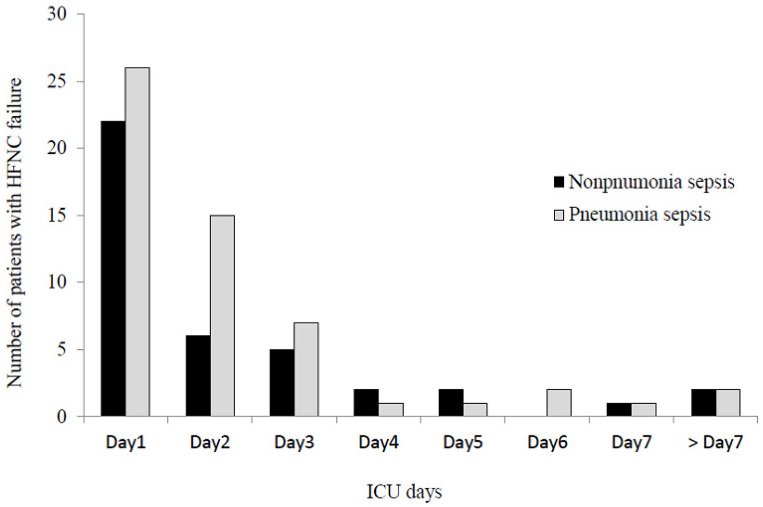
Daily number of patients experiencing high-flow nasal cannula failure. HFNC: high-flow nasal cannula; ICU: intensive care unit.

The completion rate of the 3-h sepsis bundle was 25.7%, and adequate antibiotics were administered to 88.8% of patients (Table 1). Steroid therapy and CRRT were required by 30.6% and 18.4% of patients, respectively, during the first 3 ICU days. The intubation, ICU, and hospital mortality rates were 35.0%, 24.8%, and 34.0%, respectively (Table 2). In terms of the composite outcome, HFNC failure occurred in 95 (46.1%) patients during the ICU stay. Early HFNC failure (i.e., <72 h after HFNC initiation) occurred in 81 (39.3%) patients, accounting for 85.3% of all HFNC failures (Figure 2). The incidence of early HFNC failure did not differ between the pneumonia and non-pneumonia sepsis patients. Comparisons of baselines characteristics and treatments between patients with HFNC failure and those without are presented in Supplementary Tables (Tables S1 and S2 for pneumonia sepsis; Tables S3 and S4 for non-pneumonia sepsis patients).

### 3.3. Risk Factors for Early HFNC Failure

**Figure 3:**
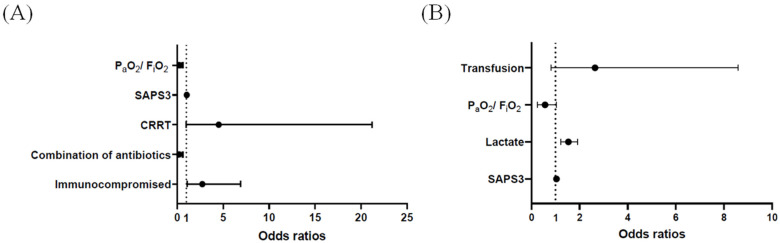
Odds ratios and 95% confidence intervals for early high-flow nasal cannula failure by multivariable analysis in (**A**) pneumonia sepsis and (**B**) non-pneumonia sepsis patients. CRRT: continuous renal replacement therapy; SAPS3: simplified acute physiology score3.

In pneumonia sepsis patients, univariable analyses revealed six variables (immunocompromised state, combination of antibiotics, CRRT, net fluid balance on day1, SAPS3, and P_a_O_2_/F_i_O_2_ ratio) associated with the early HFNC failure (*p* < 0.05). Of these, five variables were included in multivariable analysis (Table 3), and an immunocompromised state (odds ratio = (OR) 2.730; 95% confidence interval (CI): 1.082–6.889), use of an antibiotic combination (0.219; 95% CI: 0.079–0.605), and the P_a_O_2_/F_i_O_2_ ratio (0.308; 95% CI: 0.158–0.601) were significantly associated with the risk of early HFNC failure (Figure 3A). However, in non-pneumonia sepsis patients, nine variables (septic shock, steroid therapy, CRRT, transfusion, SAPS3, lactate, pH, P_a_O_2_/F_i_O_2_, and SOFA score) were associated with the early HFNC failure by univariable analyses (*p* < 0.05). Among them, six variables were initially included in multivariable analysis, and four variables were finally selected (Table 4). In the model, lactate levels (1.532; 95% CI: 1.218–1.926) were significantly associated with the risk of early HFNC failure (Figure 3B). Additionally, multivariable analysis for all enrolled patients (*n* = 206) is presented in Supplementary Table S5.

### 3.4. Rates of Early HFNC Failure According to P_a_O_2_/F_i_O_2_ Ratios and Lactate Levels

**Figure 4:**
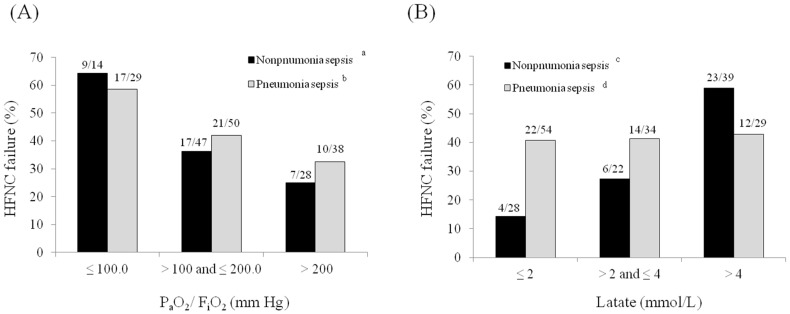
Rates of early high-flow nasal cannula failure according to (**A**) the P_a_O_2_/F_i_O_2_ ratio and (**B**) lactate level. ^a^ *p* = 0.045, ^b^ *p* = 0.028, ^c^ *p* = 0.001, and ^d^ *p* = 0.988.

Figure 4A illustrates the rates of early HFNC failure according to P_a_O_2_/F_i_O_2_ ratios (≤100 vs. >100 to ≤200 vs. >200 mm Hg). The rate of early HFNC failure increased as the P_a_O_2_/F_i_O_2_ ratio decreased in both pneumonia and non-pneumonia sepsis patients. However, when lactate levels were examined (≤2 vs. >2 to ≤4 vs. >4 mmol/L; Figure 4B), the rate of early HFNC failure did not differ in patients with pneumonia sepsis by lactate level, but was significantly higher in patients with non-pneumonia sepsis having higher lactate levels.

## 4. Discussion

This observational cohort study yielded several interesting findings. First, HFNC therapy failed in 46% of sepsis patients admitted to the ICU, and most HFNC failures occurred early (i.e., within 72 h), thus highlighting the importance of close monitoring in sepsis patients who are connected to a HFNC device (a device without alarms). Second, the predictors of HFNC failure differed between the pneumonia and non-pneumonia sepsis patients. The lactate level was a significant predictor only in the latter group.

HFNC therapy is superior to standard low-flow oxygen therapy for improving respiratory parameters (e.g., dyspnea, the respiratory rate, and oxygenation) [6,7,8], and was shown by electric impedance tomography to improve regional and global lung ventilation [21,22]. In a multicenter randomized trial, HFNC therapy was associated with lower 90-day mortality and a lower risk of intubation than non-invasive ventilation or standard oxygenation therapy in patients with P_a_O_2_/F_i_O_2_ ratios < 200 mm Hg [5]. Post-hoc analysis also showed that HFNC therapy seemed to be better than non-invasive ventilation or standard oxygenation for immunocompromised patients [4]. However, although beneficial effects have been reported for patients with various medical conditions [23,24,25,26,27], HFNC therapy for sepsis patients at risk of hemodynamic instability has not been sufficiently evaluated. Hence, our study is clinically relevant.

Retrospective studies found that oxygenation [8,28], respiratory rate [8], SOFA score [29,30], SAPS II score [31], and vasopressor use [28,32] were associated with HFNC outcomes. Recently, age, the Glasgow Coma Scale, vasopressor use, and the number of comorbidities were found to significantly predict non-invasive respiratory therapy (non-invasive ventilation and HFNC) failure in patients with coronavirus disease 2019 (COVID-19) [33]. These studies indicate the presence of several factors, other than respiratory variables, affecting HFNC outcomes, which support our findings. We also noted that sicker patients (e.g., higher lactate levels, higher SAPS3 and/or SOFA scores, and lower P_a_O_2_/F_i_O_2_ ratios) tended to fail more the HFNC therapy. However, the unique feature of our study is that we analyzed patients with sepsis of non-pulmonary origin. Although the underlying cause of hypoxemia was unknown, these patients all suffered type IV respiratory failure, which is rarely a target for clinical studies of HFNC therapy.

We found that 46% of enrolled patients experienced HFNC failure in the ICUs; 85.3% of the failures occurred within 72 h of therapy initiation. This early failure rate seems to be higher than that of the retrospective study by Kang et al. (74.3%) [34]. Although the HFNC failure rate included both intubation and death in our study, the differences may in part be attributable to differences in patient characteristics and disease severity. In the study by Kang et al., a considerable proportion of patients had acute-on-chronic respiratory failure or post-extubation failure as the cause of HFNC therapy; only 8.3% were in septic shock. However, we could only measure HFNC therapy duration on a daily (not hourly) basis; this was a limitation of our study. Nonetheless, given the concerns about delayed intubation [34], our results emphasize that sepsis patients on HFNC should be carefully monitored for HFNC failure during the early period.

The sepsis registry cohort was not designed to investigate the effects of HFNC therapy. Thus, detailed data on HFNC settings (e.g., F_i_O_2_ and flow rates) were lacking. Additionally, the criteria for HFNC therapy and intubation were not standardized, so all decisions were at the discretion of each participating hospital. However, we included only severely ill ICU patients for whom pre-HFNC P_a_O_2_/F_i_O_2_ ratios were available. Although we did not impose an upper limit, only 17 (8.3%) patients had a value of 300–400 mm Hg; the rest had values ≤300 mm Hg, thus meeting the oxygenation criterion of ARDS. However, the main purpose of our study was to investigate the clinical outcomes of HFNC therapy and the risk factors in sepsis patients prone to hemodynamic instability. Although the hospital mortality rate (34.0%) was higher in our study than that from previous sepsis studies (14.5~25.6%) [35,36,37,38], this might be attributable to high proportions of immunocompromised or elderly patients and the inclusion of hospital-acquired infections.

Interestingly, the risk factors for early HFNC failure differed somewhat between the pneumonia and non-pneumonia sepsis groups. In the pneumonia sepsis group, as well as the P_a_O_2_/F_i_O_2_ ratio, an underlying comorbidity (an immunocompromised state) and use of antibiotic combinations were significant risk factors. Conversely, in the non-pneumonia sepsis group, the lactate level, which reflects sepsis severity, was a significant risk factor for early HFNC failure. Although the P_a_O_2_/F_i_O_2_ ratio was not significant in the multivariate analysis of non-pneumonia sepsis patients, the HFNC failure rate increased with a decrease in the P_a_O_2_/F_i_O_2_ ratio (Figure 3A). Therefore, further large-scale studies are needed to confirm this. However, one possible explanation for the difference between pneumonia and non-pneumonia sepsis patients may stem from pneumonia being a primary lung disorder; thus the severity of the disease, given the lower SAPS3/SOFA scores, may be determined by the hypoxemia levels in pneumonia sepsis, whereas in non-pneumonia sepsis, organ failure may be the major determinant of HFNC outcome. Another plausible explanation for the lack of correlation between lactate levels and HFNC failure in pneumonia sepsis is that lactate in this population might be secondary for the work of breathing and less for general tissue oxygenation. Therefore, HFNC, which might lessen the work of breathing, may make the lactate less relevant as a prognostic parameter for this device failure.

In patients who undergo noninvasive therapy for AHRF, the respiratory rate and oxygenation level are important predictors of respiratory failure and the need for intubation [9,27,33,39]. Tracking dynamic changes in these parameters during HFNC therapy is important [9,39]. However, unfortunately, we lacked follow-up data on vital signs and arterial blood gas levels. Additionally, we did not calculate the ROX index [9]. However, our target population was sepsis patients admitted to the ICU with a low P_a_O_2_/F_i_O_2_ ratio. Our focus was on HFNC outcomes and associated risk factors, and not only on respiratory parameters.

Our study had several limitations. First, it used an observational design and included a small number of patients, so it was underpowered; there may have been unidentified sources of bias. Second, smoking status and hypertension, which are the variables frequently investigated in many studies, were not collected in our study. They may have had an effect on some patients. Third, as mentioned above, the criteria for HFNC and intubation were not standardized. Hence, it is possible that the treatments differed among the patients, which may have affected the HFNC outcomes. Fourth, we lacked data on HFNC settings (i.e., F_i_O_2_ and flow rate) and other respiratory parameters (e.g., dyspnea and thoracoabdominal asynchrony); there is a possibility of inadequate settings for some patients. Fifth, all of the patients were from the same country, which limits the generalizability of the results. However, to date, data on the outcomes of HFNC for sepsis patients are very limited. These patients exhibit unique clinical characteristics, and deterioration can be rapid or unpredictable compared to patients with AHRF alone. Thus, our study is clinically relevant, and may aid the design of large-scale trials.

## 5. Conclusions

In our study, a high proportion of sepsis patients who underwent HFNC therapy in ICUs experienced HFNC failure, usually within 72 h after therapy initiation, thus emphasizing the importance of close monitoring in these patients. Furthermore, in non-pneumonia sepsis, organ failure (i.e., lactate) might serve as a prognostic marker. This is in contrast with primary pneumonia sepsis, where the degree of hypoxemia and immunocompromised state might be the determinant of HFNC failure. Future large-scale studies are warranted.

## Figures and Tables

**Table 1:** Baseline characteristics and treatments among enrolled patients (*n* = 206).

Variables	Pneumonia Sepsis (*n* = 117)	Non-Pneumonia Sepsis (*n* = 89)	*p* Value
Age, years	71.0 (63.0–78.0)	71.0 (60.5–80.0)	0.956
Gender, M/F	87/30	55/34	0.054
Underlying disease			
Cardiovascular disease	23 (19.7%)	17 (19.1%)	0.920
Cerebrovascular disease	25 (21.4%)	16 (18.0%)	0.546
Chronic liver disease	7 (6.0%)	11(12.4%)	0.108
Connective tissue disease	3 (2.6%)	1 (1.1%)	0.635
Chronic lung disease	27 (23.1%)	8 (9.0%)	0.008
Chronic kidney disease	16 (13.7%)	16 (18.0%)	0.398
Diabetes	46 (39.3%)	27 (30.3%)	0.182
Immunocompromised ^a^	40 (34.2%)	31 (34.8%)	0.932
Charlson comorbidity index	5.0 (3.0–6.5)	5.0 (3.0–6.0)	0.583
ECOG	2.0 (1.0–3.0)	2.0 (1.0–3.0)	0.907
Septic shock	27 (23.1%)	54 (60.7%)	<0.001
Sepsis origin			
Lung	117 (100.0%)	0 (0.0%)	<0.001
Abdomen	0 (0.0%)	36 (40.4%)	
Urinary	0 (0.0%)	28 (31.5%)	
Skin and soft tissue	0 (0.0%)	7 (7.9%)	
CLABSI	0 (0.0%)	4 (4.5%)	
Other systemic infections	0 (0.0%)	14 (15.7%)	
SAPS3 at ICU admission	65.0 (55.0–74.0)	69.0 (67.0–81.0)	0.005
SOFA on the day of HFNC start	7.0 (5.0–9.5)	10.0 (7.0–12.0)	<0.001
At the initiation of HFNC			
pH	7.41 (7.35–7.46)	7.38 (7.31–7.45)	0.049
P_a_CO_2_, mm Hg	32.1 (27.8–38.5)	30.1 (25.9–36.0)	0.102
P_a_O_2_/F_i_O_2_	150.0 (100.5–224.2)	164.0 (117.8–230.2)	0.218
Lactate, mmol/L	2.1 (1.2–4.0)	3.2 (1.8–6.2)	<0.001
Bacteremia	15 (12.8%)	36 (40.0%)	<0.001
Infection by MDR pathogens	21 (17.9%)	19 (21.3%)	0.541
CAI/HAI	83/34	47/42	0.008
3-h sepsis bundle ^b^	28 (23.9%)	25 (28.1%)	0.499
Adequate antibiotics	101 (86.3%)	82 (92.1%)	0.190
Combination of antibiotics	87 (74.4%)	44 (49.4%)	<0.001
Steroid treatments	36 (30.8%)	27 (30.3%)	0.947
Transfusions	27 (23.1%)	47 (52.8%)	<0.001
Noninvasive ventilation	2 (1.7%)	2 (2.2%)	0.782
CRRT	11 (9.4%)	27 (30.3%)	<0.001
Pre-ICU fluid balance, mL	749.0 (200.0–1763.8)	972.0 (305.0–2238.0)	0.389
Day1 fluid balance, mL	366.4 (−34.3–901.8)	721.0 (78.5–1680.9)	0.029

CAI: community acquired infection; CRRT: continuous renal replacement therapy; CLABSI: central line associated blood stream infection; ECOG: Eastern Cooperative Oncology Group; F: female; HAI: hospital acquired infection; HFNC: high flow nasal cannula; ICU: intensive care unit; M: male; MDR: multi-drug resistance; SAPS3: simplified acute physiology score3; SOFA: sequential organ failure assessment. ^a^ Patients with hematologic malignancy, solid cancer, or drug-induced immunosuppression. ^b^ Completion rate with the 3-h sepsis bundle components.

**Table 2:** Treatment outcomes among enrolled patients (*n* = 206).

Outcomes	Pneumonia Sepsis (*n* = 117)	Non-Pneumonia Sepsis (*n* = 89)	*p* Value
Mechanical ventilation	44 (37.6%)	28 (31.5%)	0.359
ICU death	26 (22.0%)	25 (28.1%)	0.334
Hospital death	40 (34.2%)	30 (33.7%)	0.943
HFNC failure at ICU day3	48 (41.0%)	33 (37.1%)	0.566
HFNC failure at ICU day7	53 (45.3%)	38 (42.7%)	0.709
HFNC failure during ICU stay	55 (47.0%)	40 (44.9%)	0.768
Length of ICU stay, days	6.0 (3.0–11.0)	4.0 (3.0–10.0)	0.248
Length of hospital stay, days	20.0 (10.0–30.0)	19.0 (10.0–31.5)	0.803

HFNC: high flow nasal cannula; ICU: intensive care unit.

**Table 3:** Risk factors for HFNC failure in patients with pneumonia sepsis (*n* = 117) ^a^.

Variables	Univariable Analysis		Multivariable Analysis ^d^	
	OR	95% CI	OR	95% CI
Immunocompromised ^b^	2.397	1.097–5.239	2.730	1.082–6.889
Combination of antibiotics	0.351	0.149–0.824	0.219	0.079–0.605
CRRT	4.400	1.013–17.556	4.520	0.963–21.215
SAPS3 at ICU admission	1.001	1.000–1.001	1.038	0.997–1.081
PaO_2_/FiO_2_ ratio ^c^	0.502	0.300–0.841	0.308	0.158–0.601

CI: confidence interval; CRRT: continuous renal replacement therapy; OR: odds ratio; SAPS3: simplified acute physiology score3. ^a^ HFNC failure indicates a composite outcome of intubation or ICU death on ICU day3. ^b^ Patients with hematologic malignancy, solid cancer, or drug-induced immunosuppression. ^c^ P_a_O_2_/F_i_O_2_ group (≤100.0 vs. >100.0 to 200.0 vs. >200.0 mm Hg). ^d^ Five variables (*p* < 0.05 by univariable analyses) were initially included in the multivariable model: immunocompromised, combination of antibiotics, CRRT, SAPS3, and PaO_2_/FiO_2_ group. Hosmer–Lemeshow test: chi-square = 9.457, *p* = 0.305.

**Table 4:** Risk factors for HFNC failure in patients with non-pneumonia sepsis (*n* = 89) ^a^.

Variables	Univariable Analysis		Multivariable Analysis ^c^	
	OR	95% CI	OR	95% CI
SAPS3 at ICU admission	1.074	1.033–1.117	1.040	0.990–1.092
Lactate	1.618	1.302–2.012	1.532	1.218–1.926
P_a_O_2_/F_i_O_2_ ratio ^b^	0.447	0.225–0.889	0.564	0.244–1.030
Transfusion	3.826	1.505–9.725	2.643	0.813–8.590

CI: confidence interval; OR: odds ratio; SAPS3: simplified acute physiology score3. ^a^ HFNC failure indicates a composite outcome of intubation or ICU death on ICU day3. ^b^ P_a_O_2_/F_i_O_2_ group (≤100.0 vs. >100.0 to 200.0 vs. >200.0 mm Hg). ^c^ Six variables (*p* < 0.05 by univariable analyses) were initially included into the multivariable model: steroid therapy, CRRT (continuous renal replacement therapy), transfusion, SAPS3, lactate, and P_a_O_2_/F_i_O_2_ ratio. Hosmer–Lemeshow test; chi-square = 10.427 and *p* = 0.236.

## Data Availability

Sunghoon Park had full access to all the data in the study and takes responsibility for the integrity of the data and the accuracy of the data analysis. Data can be obtained from the corresponding author: Sunghoon Park (f2000tj@gmail.com).

## Supplementary Materials

The following are available online at https://www.mdpi.com/article/10.3390/jcm10163587/s1, Failure of high flow nasal cannula therapy failure in pneumonia, and Definitions for “Time Zero” and Compliance with Sepsis Bundles, Table S1. Baseline characteristics and treatments among patients with pneumonia sepsis (*n* = 117), Table S2. Severity of illness and laboratory parameters among patients with pneumonia sepsis (*n* = 117), Table S3. Baseline characteristics and treatments among patients with non-pneumonia sepsis (*n* = 89), Table S4. Severity of illness and laboratory parameters among non-pneumonia sepsis (*n* = 89), Table S5. Risk factors for HFNC failure among all enrolled patients (*n* = 206).
