# Supplementary material for: Failure of High-Flow Nasal Cannula Therapy in Pneumonia and Non-Pneumonia Sepsis Patients: A Prospective Cohort Study
Source: J Clin Med. 2021 Aug 15;10(16):3587. doi: 10.3390/jcm10163587 (PMC8396877; doi:10.3390/jcm10163587)
Supplement: Supplementary file 1 [file jcm-10-03587-s001.zip › jcm-1293732-supplementary.pdf]

Supplementary Materials

Definitions for "Time Zero" and Compliance with Sepsis Bundles

1. Time Zero[1]

In patients who presented to the emergency departments (EDs), the "time zero" was defined as the time of triage in the ED. However, in patients who were hospitalized in general wards, the "time zero" was defined as the time of sepsis recognition by physicians or nurses.

2. The 3 hour-Sepsis Bundle[2]

1) 'Compliance with the 3-hour sepsis bundle' was defined as the achievement of the bundle elements within 3 hours from the time zero.

① Measurement of lactate

② Blood culture prior to antibiotic administration

③ Administration of broad-spectrum antibiotics

④ Infusion of any bolus crystalloid fluid

References

1. Levy MM, Evans LE, Rhodes A: The Surviving Sepsis Campaign Bundle: 2018 update. Intensive Care Med 2018; 44: 925-8

2. Rhodes A, Evans LE, Alhazzani W, et al: Surviving Sepsis Campaign: International Guidelines for Management of Sepsis and Septic Shock: 2016. Crit Care Med 2017; 45: 486-552

Supplementary Table S1. Baseline characteristics and treatments among patients with pneumonia sepsis (*n* = 117)

Variables	HFNC Failure (–)[a] (*n* = 69)	HFNC Failure (+)[a] (*n* = 48)	*p* Value
Age, years	72.0 (65.5–78.5)	70.5 (62.0–77.5)	0.336
Gender, M/F	52/17	35/13	0.766
Underlying disease			
Cardiovascular disease	14 (20.3%)	9 (18.8%)	0.837
Cerebrovascular disease	17 (24.6%)	8 (16.7%)	0.301
Chronic liver disease	6 (8.7%)	1 (2.1%)	0.237
Connective tissue disease	1 (1.4%)	2 (4.2%)	0.567
Chronic lung disease	15 (21.7%)	12 (25.0%)	0.680
Chronic kidney disease	11 (15.9%)	5 (10.4%)	0.392
Diabetes	26 (37.7%)	20 (41.7%)	0.664
Immunocompromised[b]	18 (26.1%)	22 (45.8%)	0.027
Charlson comorbidity index	5.0 (3.0–7.0)	5.0 (4.0–6.0)	0.975
ECOG	2.0 (1.0–3.0)	2.0 (1.0–3.0)	0.932
Septic shock	14 (20.3%)	13 (27.1%)	0.391

Bacteremia	10 (14.5%)	5 (10.4%)	0.517
Infection by MDR pathogens	13 (18.8%)	8 (16.7%)	0.763
CAI/HAI	52/17	31/17	0.207
3-h sepsis bundle[c]	16 (32.2%)	12 (25.0%)	0.821
Adequate antibiotics	60 (87.0%)	41 (85.4%)	0.812
Combination of antibiotics	57 (82.6%)	30 (62.5%)	0.014
Steroid treatments	17 (24.6%)	19 (39.6%)	0.085
Transfusion	15 (21.7%)	12 (25.0%)	0.680
Noninvasive ventilation	2 (2.9%)	0 (0.0%)	0.512
CRRT	3 (4.3%)	8 (16.7%)	0.025
Pre-ICU net fluid balance, mL	830.0 (150.0–2010.0)	700.0 (200.0–1322.0)	0.511
Day1 net fluid balance, mL	240.0 (-120.0–682.4)	612.0 (90.3–1467.5)	0.008

CAI: community acquired infection; CRRT: continuous renal replacement therapy; ECOG: Eastern Cooperative Oncology Group; F: female; M: male; MDR: multi-drug resistance; HAI: hospital acquired infection; ICU: intensive care unit. [a]HFNC failure indicates a composite outcome of intubation or ICU death on ICU day3. [b]Patients with hematologic malignancy, solid cancer or drug-induced immunosuppression. [c]Completion rate with the 3-h sepsis bundle components.

Supplementary Table S2. Severity of illness and laboratory parameters among patients with pneumonia sepsis (*n* = 117)

**Variables**	**HFNC Failure (-)[a] (*n* = 69)**	**HFNC Failure (+)[a] (*n* = 48)**	***p* Value**
Systolic blood pressure, mm Hg	100.0 (84.5 – 124.5)	99.0 (85.3 – 142.0)	0.636
Heart rate, beats/min	109.0 (90.5 – 124.5)	108.5 (97.3 – 126.8)	0.636
Respiratory rate, breaths/min	24.0 (22.0 – 28.0)	24.5 (22.0 – 29.5)	0.748
SAPS3 at ICU admission	63.0 (52.0 – 71.0)	69.0 (61.0 – 75.0)	0.007
WBC, 10^3/uL	10.9 (7.8 – 17.3)	8.7 (4.7 – 16.7)	0.137
Hemoglobin, g/dL	10.9 (8.9 – 12.9)	10.2 (8.8 – 12.6)	0.381
Platelet, 10^3/uL	203.0 (131.0 – 290.5)	153.5 (102.5 – 258.5)	0.101
Lactate, mmol/L	2.1 (1.1 – 4.0)	2.2 (1.4 – 4.1)	0.528
Bilirubin, mg/dL	0.9 (0.5 – 1.3)	0.8 (0.6 – 1.6)	0.596
C-reactive protein, mg/dL	12.1 (5.7 – 20.9)	14.1 (7.4 – 22.3)	0.333
Albumin, g/dL	3.0 (2.7 – 3.5)	3.1 (2.5 – 3.6)	0.844
BUN, mg/dL	30.9 (21.0 – 52.7)	24.1 (13.6 – 43.7)	0.140
Creatinine, mg/dL	1.4 (0.9 – 2.2)	1.2 (0.7 – 1.8)	0.163
[Na^+], mmol/L	135.0 (133.0 – 139.0)	134.0 (127.5 – 139.0)	0.374
[K^+], mmol/L	4.2 (3.7 – 4.9)	4.0 (3.5 – 4.6)	0.289
[Cl^-], mmol/L	102.0 (97.0 – 106.0)	99.0 (92.0 – 107.0)	0.156
pH	7.41 (7.35 – 7.46)	7.41 (7.33 – 7.46)	0.899
P_aCO_2, mmHg	31.4 (26.9 – 38.5)	32.3 (28.1 – 38.8)	0.866
P_aO_2/ F_iO_2	175.2 (124.0 – 243.9)	119.9 (74.9 – 165.0)	0.001
SOFA on the day of HFNC start	7.0 (4.5 – 8.0)	8.0 (5.0 – 11.0)	0.066

BUN: blood urea nitrogen; ICU: intensive care unit; SAPS3: simplified acute physiology score3; SOFA: sequential organ failure assessment; WBC: white blood cells.[a]HFNC failure indicates a composite outcome of intubation or ICU death on ICU day3.

Supplementary Table S3 Baseline characteristics and treatments among patients with non-pneumonia sepsis (n = 89)

Variables	HFNC Failure (–)[a] (n = 56)	HFNC Failure (+)[a] (n = 33)	p Value
Age, years	70.5 (61.0–79.5)	72.0 (60.5–81.0)	0.769
Gender, M/F	33/23	22/11	0.468
Underlying disease			
Cardiovascular disease	14 (25.0%)	3 (9.1%)	0.665
Cerebrovascular disease	13 (23.2%)	3 (9.1%)	0.094
Chronic liver disease	7 (12.5%)	4 (12.1%)	0.958
Connective tissue disease	0 (0.0%)	1 (3.0%)	0.371
Chronic lung disease	5 (8.9%)	3 (9.1%)	0.979
Chronic kidney disease	11 (19.6%)	5 (15.2%)	0.594
Diabetes	18 (32.1%)	9 (27.3%)	0.629
Immunocompromised[b]	17 (30.4%)	14 (42.4%)	0.248
Charlson comorbidity index	5.0 (4.0–6.0)	5.0 (3.0–6.0)	0.911
ECOG	2.0 (1.0–4.0)	1.0 (0.0–3.0)	0.041
Septic shock	28 (50.0%)	26 (78.8%)	0.007
Bacteremia	24 (42.9%)	12 (36.4%)	0.547
Infection by MDR pathogens	13 (23.2%)	6 (18.2%)	0.576
CAI/HAI	30/26	17/16	0.851
3-h sepsis bundle[c]	14 (25.0%)	11 (33.3%)	0.398
Adequate antibiotics	52 (92.9%)	30 (90.9%)	0.707
Combination of antibiotics	30 (53.6%)	14 (42.4%)	0.310
Steroid treatments	12 (21.4%)	15 (45.5%)	0.017
Transfusion	23 (41.4%)	24 (72.7%)	0.004
CRRT	10 (17.9%)	17 (51.5%)	0.001
Noninvasive ventilation	1 (1.8%)	1 (3.0%)	1.000
Pre-ICU net fluid balance, ml	955.7 (252.5–2035.5)	1505.0 (364.8–2693.5)	0.361
Day1 net fluid balance, ml	500.0 (0.0–1416.3)	922.0 (336.9–2191.6)	0.124

CAI: community acquired infection; CRRT: continuous renal replacement therapy; ECOG: Eastern Cooperative Oncology Group; F: female; M: male; MDR: multi-drug resistance; HAI: hospital acquired infection; ICU: intensive care unit. [a]HFNC failure indicates a composite outcome of intubation or ICU death on ICU day3. [b]Patients with hematologic malignancy, solid cancer or drug-induced immunosuppression. [c]Completion rate with the 3-h sepsis bundle components.

Supplementary Table S4. Severity of illness and laboratory parameters among non-pneumonia sepsis (*n* = 89)

Variables	HFNC Failure (-)[a] (*n* = 56)	HFNC Failure (+)[a] (*n* = 33)	*p* Value
Systolic blood pressure, mm Hg	88.5 (71.0–100.8)	84.0 (71.0–92.0)	0.329
Heart rate, beats/min	108.0 (86.0–117.5)	108.0 (87.5–131.0)	0.510
Respiratory rate, breaths/min	22.5 (20.0–28.8)	24.0 (22.0–28.0)	0.272
SAPS3 at ICU admission	65.0 (57.0–75.5)	76.0 (68.5–87.0)	<0.001
WBC, 10^3/uL	9.2 (4.8–16.5)	10.5 (5.7–21.6)	0.619
Hemoglobin, g/dL	11.0 (9.9–12.5)	10.0 (8.7–11.9)	0.076
Platelet, 10^3/uL	149.0 (93.0–233.0)	127.0 (65.5–226.5)	0.646
Lactate, mmol/L	2.6 (1.5–4.3)	6.2 (3.3–9.4)	<0.001
Bilirubin, mg/dL	0.8 (0.6–2.3)	0.8 (0.6–1.8)	0.317
C-reactive protein, mg/dL	11.5 (5.3–22.5)	15.4 (8.7–24.9)	0.317
Albumin, g/dL	2.7 (2.4–3.3)	2.6 (2.2–3.3)	0.378
BUN, mg/dL	34.0 (22.4–50.9)	35.0 (20.5–51.6)	0.769
Creatinine, mg/dL	1.7 (0.9–3.3)	1.9 (0.9–3.4)	0.872
[Na^+], mmol/L	135.0 (131.0–140.0)	134.0 (131–137.5)	0.436
[K^+], mmol/L	4.0 (3.5–4.5)	4.1 (3.5–4.6)	0.502
[Cl^-], mmol/L	103.0 (98.2–106.8)	99.0 (93.5–107.5)	0.361
pH	7.41 (7.35–7.46)	7.32 (7.22–7.41)	0.001
P_aCO_2, mm Hg	32.2 (26.7–35.3)	28.0 (24.6–37.0)	0.143
P_aO_2/ F_iO_2	171.3 (135.0–251.6)	148.0 (88.6–180.4)	0.017
SOFA on the day of HFNC start	8.5 (6.3–11.0)	11.0 (8.5–13.5)	0.003

BUN: blood urea nitrogen; ICU: intensive care unit; SAPS3: simplified acute physiology score3; SOFA: sequential organ failure assessment; WBC: white blood cells. [a]HFNC failure indicates a composite outcome of intubation or ICU death at ICU day3.

Supplementary Table S5. Risk factors for HFNC failure among all enrolled patients (*n* = 206)[a]

Variables	Univariable Analysis		Multivariable Analysis[d]	
	**OR**	**95% CI**	**OR**	**95% CI**
Immunocompromised[b]	2.057	1.144–3.700	2.226	1.134–4.372
CRRT	3.846	1.830–8.086	2.776	1.163–6.626
Steroid therapy	2.395	1.306–4.390	2.314	1.154–4.640
Combination of antibiotics	0.519	0.291–0.928	0.486	0.247–0.956
Lactate	1.236	1.104–1.384	1.149	1.019–1.295
P_aO_2/ F_iO_2 ratio[c]	0.479	0.317–0.723	0.414	0.259–0.662

CI: confidence interval; CRRT: continuous renal replacement therapy; OR: odds ratio; [a]HFNC failure indicates a composite outcome of Intubation or ICU death on ICU day3. [b]Patients with hematologic malignancy, solid cancer or drug-induced immunosuppression. [c]P_aO_2/ F_iO_2 group (≤ 100.0 vs. > 100.0 to 200.0 vs. > 200.0 mm Hg). [d]Eleven variables ($p < 0.05$ by univariable analyses) were initially included in the multivariable model: immunocompromised, cerebrovascular disease, septic shock, lactate, P_aO_2/ F_iO_2 ratio, SAPS3 (simplified acute physiology score3), SOFA (sequential organ failure assessment), combination of antibiotics, steroid therapy, CRRT, and transfusion. Hosmer-Lemeshow test; chi-square = 3.071 and $p = 0.930$.
